# Lactate transport facilitates neurite outgrowth

**DOI:** 10.1042/BSR20180157

**Published:** 2018-10-02

**Authors:** Kun Chen, Peng Cheng, Huan Wang, Shutao Gao, Xiao Li, Zhenhan Deng, Jian Liu, Xuying Sun

**Affiliations:** 1 Biological Engineering and Regenerative Medicine Center, Tongji Hospital, Tongji Medical College, Huazhong University of Science and Technology, Wuhan 430030, China; 2 Department of Orthopedics, Tongji Hospital, Tongji Medical College, Huazhong University of Science and Technology, Wuhan 430030, China; 3 Department of Orthopedics, Xiangya Hospital, Central South University, Changshan 410000, China

**Keywords:** Akt, Glycogen synthase kinase 3β (GSK-3β), L-lactate, Monocarboxylate transporters-2 (MCT-2), Neurite outgrowth

## Abstract

How glia affect neurite outgrowth during neural development has not been well elucidated. In the present study, we found that disruption of lactate production using 1,4-dideoxy-1,4-imino-D-arabinitol (DAB) and isofagomine significantly interfered with neurite outgrowth and that exogenous application of L-lactate rescued neurite growth failure. Monocarboxylate transporter-2-knockout, which blocked the lactate shuttle in neurons, showed a remarkable decrease in the length of axons and dendrites. We further demonstrated that Akt activity was decreased while glycogen synthase kinase 3β (GSK3β) activity was increased after astrocytic glycogen phosphorylase blockade. Additionally, GSK3βSer9 mutation reversed neurite growth failure caused by DAB and isofagomine. Our results suggested that lactate transportation played a critical role in neural development and disruption of the lactate shuttle in quiescent condition also affected neurite outgrowth in the central nervous system.

## Introduction

Lactate transport is an effective method for neurons in the brain to utilize energy. Glucose is an important source of energy for the brain, which travels through the blood–brain barrier and enters neurons and astrocytes via transporters. Astrocytes can store glycogen as well as a certain amount of glucose. The stored glucose and the astrocyte-produced lactate would be capable of being exported to neurons as fuel through monocarboxylate transporters (MCTs) located on the membranes of glia and neurons [1,2]. Glia are believed to give mainly structural and metabolic support to neurons in the central nervous system [3]. However, growing evidence suggests that astrocytes may also play more active roles, especially during robust neuronal activity. Murphy et al. identified elevated Ca^2+^ from astrocytes in response to neuronal activity [4]. Thus, astrocytes are able to regulate neural and synaptic plasticity [5] and influence information process [6]. Recently, the critical role of astrocytes in learning and memory has been investigated by many researchers and labs. They suggest that L-lactate released by astrocytes [1,2] is vital to long-term memory formation [7] and the induction of the expression of some synaptic plasticity-related genes like *Arc, c-Fos*, and *Zif268* [8]. In another work, Lee et al. found that lactate transport through oligodendroglia and neurons supports axon survival and function, which indicates that the lactate shuttle might play a certain role in axon growth [9]. The exchange of metabolic intermediates between neurons and glia becomes obvious during robust neuronal activity. The reason for activity dependence might be that high metabolic demands are required in synaptic transmission. Nevertheless, little is known about the role of energy transfer in quiescent condition.

Neurite outgrowth impairment has been reported in many neuronal disorder diseases, like amyotrophic lateral sclerosis (ALS) [10] and Alzheimer’s disease [11]. In the present study, we investigated whether disruption of the astrocyte–neuron lactate shuttle would interfere with the outgrowth and development of axons and dendrites. Results from the present study might provide new evidence for early-stage intervention in the treatment of neurodegenerative diseases.

## Materials and methods

### Antibodies and reagents

1,4-Dideoxy-1,4-imino-D-arabinitol (DAB), L-lactate, isofagomine, and internal control antibody anti-α-Tubulin mouse mAb (DM1A,1:5000) were purchased from Sigma (St. Louis, MO, U.S.A.). Primary antibodies of phosphorylated glycogen synthase kinase 3β (GSK3β) at Ser-9 (pS9, 1:1000) and phosphorylated Akt at Ser-473 (p-Akt, 1:1000) were purchased from Cell Signaling Technology (Beverly, MA, U.S.A.). Antibodies used to identify axons and dendrites, tau-1 (1:200), and antimicrotubule-associated protein-2 (MAP-2, 1:200) were purchased from Millipore (Billerica, MA, U.S.A.). Anti-GFAP antibody was purchased from Abcam (Cambridge, UK). Cell Counting Kit-8 (CCK-8) was purchased from Dojindo Molecular Technologies, Japan. Neurobasal and B27 were purchased from Invitrogen (Grand Island, NY, U.S.A.)

### Primary neuronal culture and fluorescence imaging

Primary cortical neurons were cultured from embryonic E16–18 Sprague-Dawley rats as previously reported [12]. First, we dissected the cortical brains from the embryos and cut the tissues into pieces, and then digested the tissues using 0.25% trypsin for 5 min. After that, the trypsin was removed and cells were plated into the plating medium containing DMEM/F12 and 10% FBS. The neurons were then cultured in an incubator with 5% CO_2_ at 37°C. After 2–4 h, the medium was changed to Neurobasal adding 2% B27 (maintenance medium). Afterward, the cells were subjected to different treatments.

At the end of each treatment, neurons were prepared for immunofluorescence. Neurons were quickly fixed with 4% paraformaldehyde for 15 min, followed by 0.1% Triton X-100 to permeate the membrane, and then 3% bovine serum albumin was applied to block nonspecific sites. Primary antibodies were applied overnight at 4°C. Alexa 488 or 543 conjugated secondary antibodies (1:1000, Invitrogen, Carlsbad, CA, U.S.A.) were incubated for 1 h. The pictures were taken using an LSM710 confocal microscope (Zeiss, Jena, Germany). The number of dendrites was quantified and the length of the axons and dendrites was calculated using ImageJ after the pictures were taken. The longest neurite was measured as an axon. The primary and secondary dendrites were quantified in the total dendrite number. For GFAP staining, DAPI was applied to stain the nucleus after secondary antibody was applied. The percentage of GFAP-positive cells was calculated as GFAP-positive cells/ DAPI-positive cells × 100%.

### CCK-8 assay

Primary neurons were seeded in a 96 well plate. At the end of treatment, 90 μl of fresh medium was changed in each well and then 10 μl of CCK-8 solution was added. After 4 h, the OD value was measured using a Biotek plate reader (Winooski, VT, U.S.A.) at 450 nm wavelength.

### Western blotting

For Western blotting, samples were collected in the loading buffer. Then the samples were boiled for 5 min at 100°C and were subjected to electrophoresis, and then the proteins were transferred to PVDF membranes. After 5% nonfat milk blockage for 1 h, the membranes were incubated with primary antibodies at 4°C overnight. Anti-mouse or rabbit secondary antibodies were applied to the membranes for 1 h at room temperature. Afterward, the membranes were developed using the Bio-Rad Chemi Doc XRS Imaging System (Hercules, CA, U.S.A.). The phosphorylated levels of specific proteins were normalized by DM1A accordingly.

### Neuron transfection

Lentivirus-LV-siMCT-2 and lentivirus-LV-ssiMCT-2 were purchased from Genechem Technologies (Shanghai, China). Lentivirus plasmids for overexpressing GSK3β and its mutant were constructed as follows: open reading frame fragments of wild-type GSK3β and mutated GSK3βS9A were amplified and cloned into pCDH-CMV-MCS-EF1-GreenPuro cDNA Cloning and Expression Vector separately and were named as LV-GSK3βwt and LV-GSK3βS9A. Then 293T cells were co-transfected with the lentiviral expression construct and packaging plasmid mix for 48 to 72 h. After that, the viral supernatants were collected and stored at −80°C until use.

Primary rat cortical neuron cultures were infected by LV-siMCT-2, LV-ssiMCT-2, LV-GSK3βwt, or LV-GSK3βS9A immediately after the plating medium was replaced. All the viruses were transfected for 72 h. In the MCT-2 silencing experiment, neurons were directly fixed after transfection. In the GSK3β mutation experiment, DAB or isofagomine was co-applied during the transfection. After 72 h, the cells were fixed and neurite length was calculated using Image J.

### Real-time quantitative PCR

After 72 h of LV-siMCT-2 and LV-ssiMCT-2 transfection, the mRNA level of monocarboxylate transporter-2 (MCT-2) was tested using RT-PCR. Total RNA was extracted with TRIzol according to the manufacturer’s instruction (Invitrogen, Carlsbad, CA, U.S.A.) and then the RNA was transcribed to cDNA using the reverse transcription kit (Takara, Dalian, China). The PCR primers used in this experiment were as follows: MCT-2 forward primer 5’-CGAAGAGACTCAGTAAGGTATCA-3’, reverse primer 5’-GCCATCACAGACAGCAAGAA-3’, glyceraldehyde-3-phosphate dehydrogenase (*GAPDH*) forward primer 5’-TTCAACGGCACAGTCAAGG-3’, reverse primer 5’- CTCAGCACCAGCATCACC-3’. The PCR cycle used in the present study was as follows: 95°C/30 s, 40 cycles of 95°C/5 s, 60°C/30 s, and 72°C/30 s; melt-curve analysis was performed after each experiment. The amplification PCR tests were performed with a StepOnePlus Real-Time PCR Detection System (Thermo Fisher, New York, NY, U.S.A.). The levels of the *MCT-2* were normalized by the *GAPDH*.

### Statistical analysis

All values in the present study were shown as mean ± S.D. error bars were presented in each histogram, indicating the S.D. in each experiment and helping to show estimated error or uncertainty to give a general sense of precision. Every experiment was performed at least three times. One-way factor ANOVA was applied for comparing among multiple groups and *t* test was employed for two groups. Changes were considered as significance at *P*<0.05.

## Results

### Disruption of astrocyte–neuron lactate transport reduced neurite outgrowth

**Figure 1:**
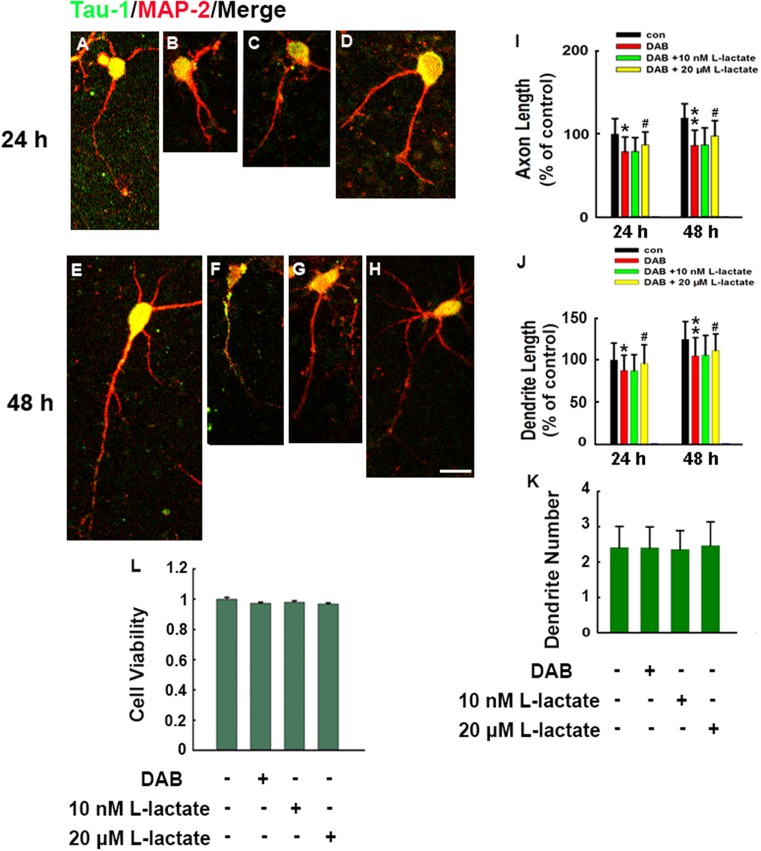
Disruption of astrocyte–neuron lactate transport caused by DAB-reduced neurite outgrowth Rat primary cortical neurons were dissected from E16–18 embryonic pups. Immediately after plating, neurons were pre-treated for different concentrations of L-lactate for 30 min and were exposed with or without an inhibitor of glycogen phosphorylation DAB (100 μM) for 24 or 48 h. After treatment, neurons were fixed and double stained with MAP-2 (red) and tau-1 (green) and merged images were presented. Representative images of (**A**) Control neuron (con 24 h), (**B**) DAB-treated neuron (24 h), (**C**) DAB-exposed and 10 nM L-lactate pre-treated neuron (24 h), (**D**) DAB-exposed and 20 μM L-lactate pre-treated neuron (24 h) and (**E**) Control neuron (con 48 h), (**F**) DAB-treated neuron (48 h), (**G**) DAB-exposed and 10 nM L-lactate pre-treated neuron (48 h), (**H**) DAB-exposed and 20 μM L-lactate pre-treated neuron (48 h), and (**I**) quantitative analysis of the axon length for 24 and 48 h exposure in (A–H) (d*f* = 244, *F* = 50.144). (**J**) Quantitative analysis of the dendrite length for 24 and 48 h in (A–H) (d*f* = 244, *F* = 60.12). (**K**) Quantification of the neurite number in neurons after 48 h exposure in each treatment. (**L**) Cell viability was measured using a CCK-8 assay. *P<0.05, **P<0.01 versus control neurons, #*P*<0.05 versus DAB-treated neurons. Approximately 60–90 neurons were calculated in each group. Scale bar: 50 μm.

To test whether elimination of the lactate produced by astrocytes affected neurite growth, we cultured neurons from E16 to E18 rat embryos using Neurobasal media, which allowed for long-term maintenance neuronal cell phenotype and growth. To prove the presence of astrocytes, we used GFAP antibody to identify astrocytes 24 h after plating. GFAP is a specific marker for astrocytes although not all astrocytes express GFAP [13,14]. Results showed 12.2% of the cells were GFAP positive in the system (Supplementary Figure S1). In the brain, lactate release from astrocytes was glycogen derived; *in vitro* studies have suggested that glycogenolysis results in lactate release [1,2]. We therefore investigated the function of the lactate shuttle by disrupting glycogen phosphorylation. Immediately after plating, an inhibitor for glycogen phosphorylation, 100 μM DAB [7,15,16], was applied to treat the neurons for 24 and 48 h. After treatment, tau-1 and MAP-2 double fluorescence staining was performed to identify axons and dendrites, respectively [17]. MAP-2 was reported to localize at dendrites and proximal axons, whereas tau-1, as a microtubule-associated protein, mainly localized at axons. Merged images were showed in the study. At the 24 and 48 h timepoints, the longest axon and dendrite were co-localized, which indicated slower axon outgrowth at 24 and 48 h. DAB significantly suppressed the length of neuron axons and dendrites at 24 (Figure 1A,B,I,J) and 48 h (Figure 1E,F,I,J), while the dendrite number was not affected (Figure 1K).

Blockage of glycogenolysis significantly reduced lactate transportation between astrocytes and neurons. We next asked whether the DAB-induced neurite growth failure could be reversed by the exogenous application of lactate. Half an hour before DAB exposure, 10 nM (Figure 1C,G,I,J) and 20 μM (Figure 1D,H,I,J) L-lactate was used to treat the neurons for 24 and 48 h. Our data showed that 10 nM lactate did not reverse the DAB-induced neurite growth failure, while 20 μM lactate significantly restored neurite growth retention. We then used CCK-8 assays to analyze the cell viability, which showed that DAB-exposed neurons exhibited almost equal viability compared with control neurons. Likewise, 10 nM and 20 μM L-lactate pre-incubation had no impact on neuron viability (Figure 1L).

**Figure 2:**
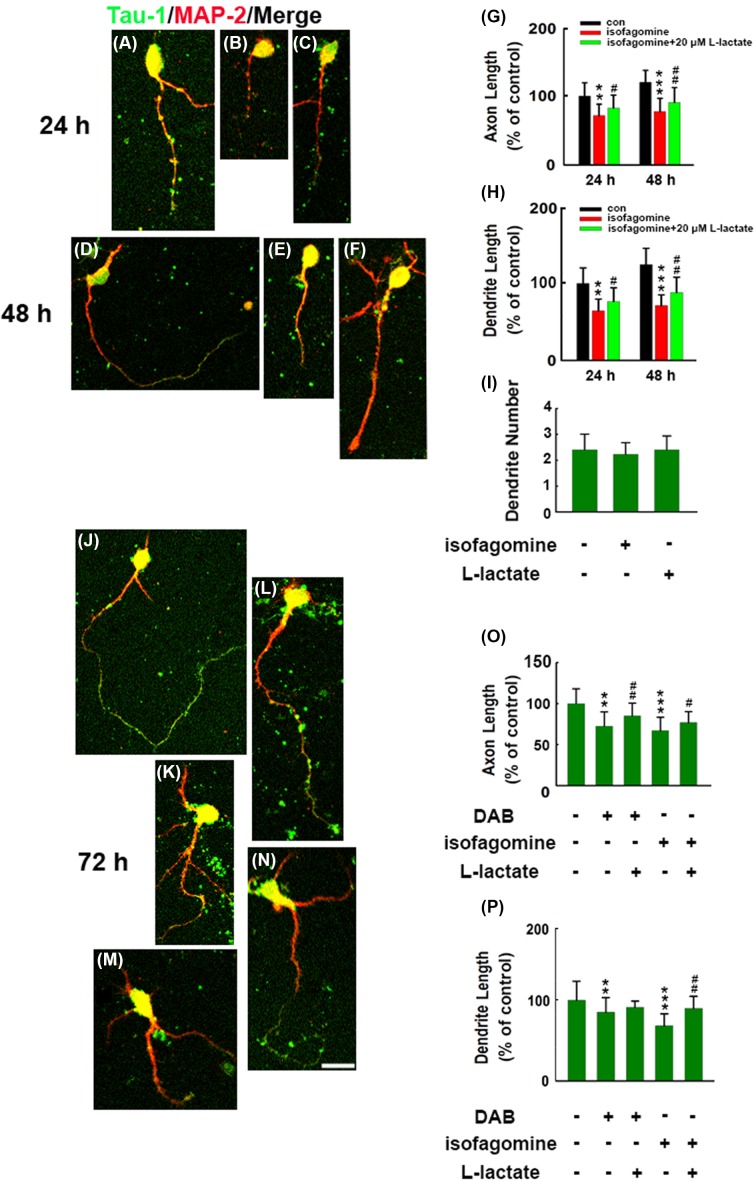
Disruption of astrocyte–neuron lactate transport caused by isofagomine-reduced neurite outgrowth Rat primary cortical neurons were dissected from E16–18 embryonic pups. Immediately after plating, neurons were pre-treated with 20 μM of L-lactate for 30 min and were exposed with or without a glycogen phosphorylase inhibitor, isofagomine (8 μM), for 24 or 48 h. After treatment, neurons were fixed and double stained with MAP-2 (red) and tau-1 (green) and merged images were presented. Representative images of (**A**) control neuron (con 24 h), (**B**) isofagomine-treated neuron, (**C**) isofagomine-exposed and 20 µM L-lactate pre-treated neuron and for 48 h (**D**) control neuron (con 48 h), (**E**) isofagomine-treated neuron (48 h), (**F**) isofagomine-exposed and 20 μM L-lactate pre-treated neuron (48 h). (**G**) Quantitative analysis of the axon length in (A–F) (d*f* = 281, *F* = 70.18). (**H**) Quantitative analysis of the dendrite length in (A–F) (d*f* = 281, *F* = 57.36). (**I**) Quantification of the neurite number in neurons after 48 h exposure in each treatment. **P<0.01, ***P<0.001 versus control neurons, #*P*<0.05, ##*P*<0.01 versus isofagomine-treated neurons. Neurons were exposed with or without a glycogen phosphorylase inhibitor, DAB (100 μM) or isofagomine (8 μM), and were pre-treated with 20 μM of L-lactate for 72 h. (**J**) Control neuron (con, 72 h). (**K**) DAB-treated neurons (72 h). (**L**) Twenty micromolar L-lactate pre-treated before DAB-exposed neuron (72 h). (**M**) Isofagomine-treated neuron (72 h). (**N**) Twenty micromolar L-lactate pre-treated before isofagomine-exposed neuron (72 h). (**O**) Quantitative analysis of the axon length in (J–N) (d*f* = 292, *F* = 59.16). (**P**) Quantitative analysis of the dendrite length in (J–N) (d*f* = 292, *F* = 75.33). **P<0.01, ***P<0.001 versus con neurons, #*P*<0.05, ##*P*<0.01 versus DAB- or isofagomine-treated cells. Approximately 60–90 neurons were calculated in each group. Scale bar: 50 μm.

Similarly, we used another glycogen phosphorylase inhibitor isofagomine [18] to treat the neurons. When 8 μM of isofagomine was applied to neurons for 24 and 48 h, the length of axons and dendrites remarkably decreased (Figure 2A,B,D,E,G,H), whereas the dendrite number was not altered (Figure 2I). As expected, 20 μM lactate pre-incubation reinstated the neurite growth failure caused by isofagomine (Figure 2C,F,G,H). We further extended to 72 h and at this timepoint, tau-1 (green) outgrew to MAP-2 (red) in control neurons, suggesting that axons underwent much faster growth at 72 h. We found that both DAB and isofagomine incubation significantly reduced axon and dendrite growth (Figure 2J,K,M,O,P); meanwhile, axon tau-1 staining still overlapped with dendrite MAP-2 after treatment. However, L-lactate pre-incubation reversed the isofagomine-induced dendrite growth failure (Figure 2L,N,O,P). Longer tau-1-stained axons were observed in L-lactate co-applied neurons (Supplementary Table S1).

### MCT-2 knockout induced neurite outgrowth failure

**Figure 3:**
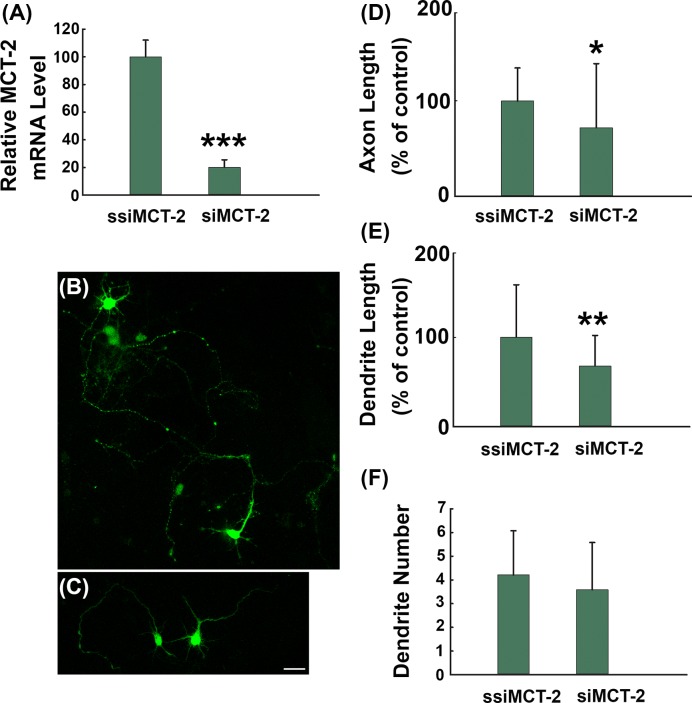
MCT-2 knockout induced neurite outgrowth failure Rat cortical neurons were transfected with scrambled control LV-ssiMCT-2 or MCT-2 knockout virus LV-siMCT-2 12 h after seeding. (**A**) After 72 h of transfection, the total RNA was extracted from the neurons, and the MCT-2 mRNA level was detected using RT-PCR. Or the neurons were fixed and the representative micrographs of neurons were showed in (**B**) LV-ssiMCT-2 transfected neuron and (**C**) LV-siMCT-2 transfected neuron. (**D**) Quantitative analysis of the axon length in (B) and (C) (*t* = −2.5666, d*f* = 70.367). (**E**) Quantitative analysis of the dendrite length in (B) and (C) (*t* = −2.695, d*f* = 74.964). (**F**) Quantification of neurite number in neurons after transfection. *P<0.05, **P<0.01, ***P<0.001 versus LV-ssiMCT-2 transfected neurons. Approximately 60 neurons were quantified in each group. Scale bar: 50 μm.

In the central nervous system, lactate transport occurs via MCTs. MCTs are mainly located at the membrane and act as carriers which can transport monocarboxylates such as lactate, pyruvate, and ketone bodies out of the cell membrane. MCT4 is expressed mainly by astrocytes, MCT2 is mainly neuronal, and MCT1 is localized on astrocytes, endothelial cells of microvessels, ependymocytes, and oligodendrocytes [19]. As a result, MCT-2 knockout could efficiently disrupt lactate transportation into neurons. To further explore the role of lactate transportation in neurite outgrowth, MCT-2 si-RNA were generated and fused with the lentivirus expressing EGFP, hereafter named LV-siMCT-2, or the scrambled control (LV-ssiMCT-2). The knockout efficiency was tested by PCR. The expression level of MCT-2 mRNA in cells transfected with LV-siMCT-2 was ~20% of that in LV-ssiMCT-2 transfected cells (Figure 3A). To detect the role of MCT-2 in neurite outgrowth failure, we transfected neurons with the two lentivirus constructs above for 72 h. After that, neurons were fixed and the length of axons and dendrites was estimated. As shown in Figure 3B–E, reduced length of axons and dendrites was found in LV-siMCT-2 transfected neurons compared with LV-ssiMCT-2 transfected neurons. Additionally, there was no significant difference in dendrite number (Figure 3F and Supplementary Table S2).

### Disruption of lactate transport leads to neurite outgrowth failure through Akt-GSK3β pathway

**Figure 4:**
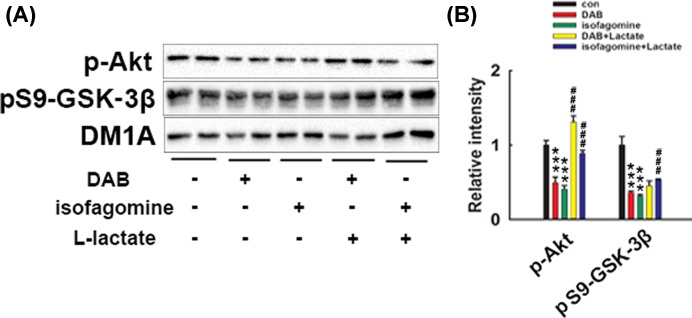
Western blot analysis of the phosphorylated levels of Akt and Ser9 (S9) of GSK3β in DAB- or isofagomine-treated neurons Rat primary cortical neurons were treated with DAB (100 μM) or isofagomine (8 μM) for 30 min, with or without L-lactate (20 μM) pre-treatment. After treatment, cell lysates were collected and subjected to Western blots. (**A**) Phosphorylated levels of Akt and Ser9 (S9) of GSK3β were detected. (**B**) Quantitative analysis of the blots in (A). Phosphorylated Akt and GSK3β levels were normalized with DM1A. ***P<0.001 versus con neurons, ###*P*<0.001 versus DAB- or isofagomine-treated neurons, d*f* = 8, *F* = 95.301 in the DAB-treated group; d*f* = 8, *F* = 121.839 in isofagomine-treated neurons.

Akt has been revealed to be an important regulator of many aspects of neurite outgrowth, such as elongation, branching, and caliber [20]. The downstream GSK3β plays a critical role in neuronal polarity formation and turning neurites into axons in the central nervous system (CNS) [21,22]. To analyze the underlying mechanisms, Western blot was applied to test the protein levels of Akt and GSK3β in neuron cultures. Twenty minutes after DAB and isofagomine incubation, the levels of p-Akt at Ser473 and the inactive form of phosphorylated Ser9 (pS9) of GSK3β were both significantly reduced (Figure 4A,B), which indicated Akt activity was down-regulated while GSK3β activity was enhanced when lactate production was blocked. However, lactate pre-incubation reversed the p-Akt level in both isofagomine- and DAB-treated neurons. Interestingly, pre-treated lactate reversed the pS9-GSK3β level in isofagomine-exposed neurons but not in DAB-treated neurons (Figure 4A,B).

**Figure 5:**
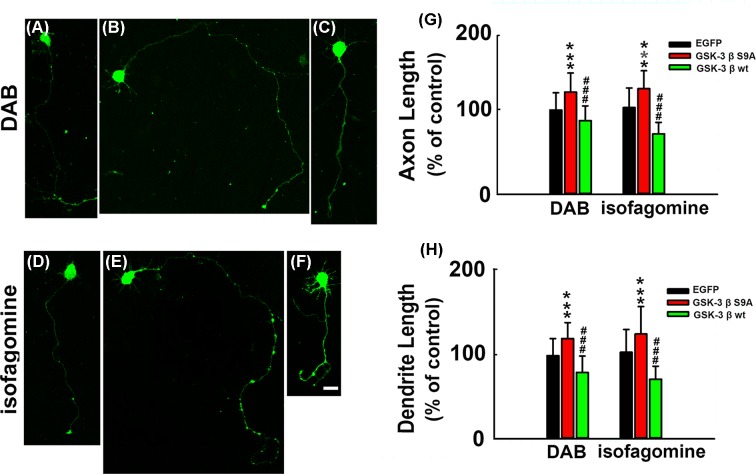
GSK3β plays a key role in neurite growth failure induced by lactate shuttle disruption Rat cortical neurons were transfected with lentivirus suspensions of empty vector, LV-GSK3βS9A or LV-GSK3βwt, 12 h after seeding; DAB (100 μM) and isofagomine (8 μM) were added to the medium, respectively. After 72 h, cells were fixed and representative images of neurons were showed in DAB-incubated neurons, (**A**) empty vector transfected neuron. (**B**) GSK3βS9A transfected neuron. (**C**) GSK3βwt transfected neurons. And in isofagomine-incubated neurons, (**D**) empty vector transfected neuron. (**E**) GSK3βS9A transfected neuron. (**F**) GSK3βwt transfected neurons. (**G**) Quantitative analysis of the axon length in (A–F) (d*f* = 227, *F* = 48.697 in the DAB-treated group; d*f* = 221, *F* = 105.641 in the isofagomine-treated group). (**H**) Quantitative analysis of the dendrite length in (A–F) (d*f* = 227, *F* = 73.202 in the DAB-treated group; d*f* = 221, *F* = 61.492 in the isofagomine-treated group). ***P<0.001 versus empty vector transfected neurons, ###*P*<0.01 versus GSK3βS9A transfected neurons. In each group, ~50–90 neurons were calculated. Scale bar: 50 μm.

To further verify whether GSK3β plays a central role in neurite growth failure induced by lactate shuttle disruption, a plasmid with mutated Ser 9 to Ala (GSK3βS9A) was used to inactivate GSK3β. This mutation in Ser9 was reported to significantly inactivate GSK3β [23]. We transfected primary cultured neurons using lentivirus suspensions of LV-GSK3βwt and LV-GSK3βS9A. DAB and isofagomine were added into the medium respectively at the same time of the transfection process and was further incubated for 72 h. Our data showed GSK3βS9A transfected neurons with inactivated GSK3β exhibited significantly longer axon and dendrite length than the empty vector and wild-type GSK3β transfected neurons in both DAB- and isofagomine-treated neurons. These results indicated that Akt/GSK3β was indeed involved in lactate deficiency induced neurite outgrowth failure. (Figure 5A–H and Supplementary Table S2).

## Discussion

Axon elongation and dendrite development are crucial processes for the establishment of a functional neuronal network. Lactate, the metabolic byproduct of glycolysis, is synthesized by astrocytes and consumed by neurons. Furthermore, synaptic activity induced glycolysis was able to facilitate membrane lipid provision and promote outgrowth [24]. Compared with resting-state glycolysis and baseline lactate production, increased brain activity was reported to remarkably increase local lactate production [25]. However, the function of the lactate shuttle in quiescent condition was not clear. We found DAB and isofagomine application, two glycogen phosphorylase inhibitors which block lactate production, significantly impaired neurite outgrowth in cultured neurons, but the use of L-lactate rescued the growth failure, which implied that the lactate shuttle might play a role in neurite elongation. In the present study, we applied a neuron-dominate model to specifically investigate the function of the neuron–glia lactate shuttle during neuronal development. *In vivo*, astrocytes were shown to be mature three to four weeks after birth as indicated by their typical morphology [26,27], so we believe that astrocytes in our system are immature. To our surprise, the results showed a significant neurite growth failure after lactate production was blocked, which suggested that immature neurons had already taken lactate as an energy substrate during embryonic development. The metabolic link between astrocytes and neurons constituted at least two important aspects. One was lactate production, whose disruption significantly affected neurite outgrowth as shown by our results, and the other was neuronal; thus MCT-2 knockout, which located at neurons and transported lactate into neurons, should also collapse the lactate shuttle between astrocytes and neurons. Our data did show that when MCT-2 was knockout, neurite outgrowth was remarkably attenuated. All these results strongly suggested that the lactate shuttle facilitated neurite outgrowth during early neuronal development. However, none of the treatments affected dendrite number; the reason may be explained by insufficient exposure time in the present study.

Among the intracellular signaling mechanisms, Akt-GSK3β signaling in the CNS has been reported to play a critical role in the regulation of neuronal maturity and network formation. [22,28,29]. To further explore the underlying mechanisms of the lactate shortage induced impairment of neurite outgrowth, we measured the protein level of the active form of p-Akt and the inactive form of phosphorylated GSK3βSer9 using Western blots. Results demonstrated that after DAB and isofagomine exposure, Akt activity decreased while GSK3β activity was enhanced and that lactate pre-incubation partially restored the activity of Akt and GSK3β. Moreover, mutation of Ser9 in GSK3β, which caused GSK3β inactivation, eliminated neurite outgrowth failure caused by DAB and isofagomine. All the data above implied that the lactate shuttle in neuron–glia metabolic coupling induced neurite outgrowth failure at least partially by targeting the Akt-GSK3β pathway. The metabolic link within the neuron and glia not only influences learning and memory, but also plays a central role in neuron development and neuronal network formation.

## Supporting information

**Fig 1:** GFAP staining in primary rat cortical neurons culture.

**Supplementary Table 1:** Axon and dendrite length after different treatments

**Supplementary Table 2:** Axon and dendrite length 72 h after transfection

## Abbreviations

ALS: amyotrophic lateral sclerosis
DAB: 1,4-dideoxy-1,4-imino-D-arabinitol
GSK3β: glycogen synthase kinase 3β
mAb: monoclonal antibody
GFAP: glial fibrillary acidic protein
DMEM: Dulbecco’s Modified Eagle Medium
