# Supplementary material for: Lactate transport facilitates neurite outgrowth
Source: Biosci Rep. 2018 Oct 2;38(5):BSR20180157. doi: 10.1042/BSR20180157 (PMC6167502; doi:10.1042/BSR20180157)
Supplement: Supplementary file 1 [file bsr20180157_Supp1.pdf]

Supplementary Fig 1

Fig 1, GFAP staining in primary rat cortical neurons culture.

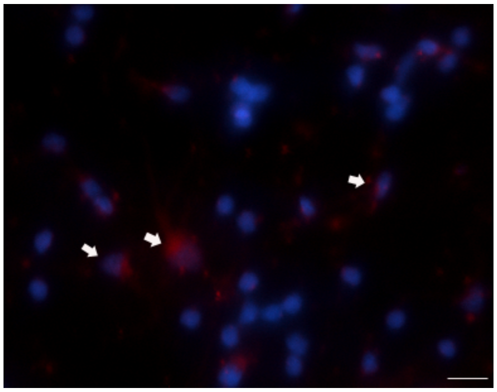

Rat primary cortical neurons were dissected from E16-18 embryonic pups. 24 h after plating, neurons were fixed and subjected to GFAP staining, arrows showed GFAP positive astrocytes, the percentage of astrocytes is about 12.2%. Scale bar: 100 μm.

Supplementary Table 1, Axon and dendrite length after different treatments

	Axon Length (μm, mean±SD)	Dendrite Length (μm, mean±SD)
Control 24 h	158.13±29.58	116.98±23.42
Control 48 h	188.96±27.21	145.58±24.58
Control 72 h	200.24±35.32	161.3±38.19
DAB 24 h	124.76±27.91	102.63±20.98
DAB+10 nM Lactate 24 h	125.43±26.32	102.34±22.1
DAB+20 μM Lactate 24 h	138.24±24.19	112.36±25.64
DAB 48 h	136.62±29.17	122.75±25.14
DAB+ 10 nM Lactate 48 h	138.1±32.11	123.72±27.21
DAB+ 20 μM Lactate 48 h	154.81±29.13	130.26±22.91
DAB 72 h	145.1±34.66	137.53±29.43
DAB+ 20 μM Lactate 72 h	170.59±30.45	147.98±12.53
Isofagomine 24 h	112.88±26.05	75.52±17.82
Isofagomine+20 μM Lactate 24 h	130.55±29.19	89.55±20.89
Isofagomine 48 h	122.23±29.86	83.25±16.46
Isofagomine+20 μM Lactate 48 h	142.92±34.14	103.41±22.95
Isofagomine 72 h	134.12±32.69	110.46±23.93
Isofagomine+20 μM Lactate 72 h	153.63±26.78	145.24±23.9

Supplementary Table 2, Axon and dendrite length 72 h after transfection

	Axon Length (μm, mean±SD)	Dendrite Length (μm, mean±SD)
EGFP (DAB)	156.29±32.01	128.5±26.3
GSK-3β S9A (DAB)	189.53±35.52	154.96±24.59
GSK-3β wt (DAB)	136.62±27.63	102.56±25.2

EGFP (isofagomine)	161.05±36.28	133.73±35.28
GSK-3β S9A (isofagomine)	196.06±33.15	161.85±42.41
GSK-3β wt (isofagomine)	112.27±21.35	92.13±20.01
ssiMCT-2	197.09±68.26	107.84±63.3
siMCT-2	141.93±132.31	73.22±36.86
